# A perspective on enhancing representative samples in developmental human neuroscience: Connecting science to society

**DOI:** 10.3389/fnint.2022.981657

**Published:** 2022-09-02

**Authors:** Kayla H. Green, Ilse H. Van De Groep, Lysanne W. Te Brinke, Renske van der Cruijsen, Fabienne van Rossenberg, Hanan El Marroun

**Affiliations:** ^1^Erasmus School of Social and Behavioural Sciences, Erasmus University Rotterdam, Rotterdam, Netherlands; ^2^Department of Child and Adolescent Psychiatry, Amsterdam University Medical Centre, Amsterdam, Netherlands; ^3^Department of Developmental and Educational Psychology, Leiden University, Leiden, Netherlands; ^4^Department of Psychology, Education and Child Studies, Erasmus School of Social and Behavioural Sciences, Erasmus University Rotterdam, Rotterdam, Netherlands; ^5^Department of Child and Adolescent Psychiatry/Psychology, Erasmus University Medical Center, Rotterdam, Netherlands

**Keywords:** neuroscience, development, representativeness, diversity, society, samples, adolescence, marginalized groups

## Abstract

Marginalized groups are often underrepresented in human developmental neuroscientific studies. This is problematic for the generalizability of findings about brain-behavior mechanisms, as well as for the validity, reliability, and reproducibility of results. In the present paper we discuss selection bias in cohort studies, which is known to contribute to the underrepresentation of marginalized groups. First, we address the issue of exclusion bias, as marginalized groups are sometimes excluded from studies because they do not fit the inclusion criteria. Second, we highlight examples of sampling bias. Recruitment strategies are not always designed to reach and attract a diverse group of youth. Third, we explain how diversity can be lost due to attrition of marginalized groups in longitudinal cohort studies. We provide experience- and evidence-based recommendations to stimulate neuroscientists to enhance study population representativeness *via* science communication and citizen science with youth. By connecting science to society, researchers have the opportunity to establish sustainable and equal researcher-community relationships, which can positively contribute to tackling selection biases.

## Introduction

Developmental neuroscientists generally aim to include representative samples in their scientific studies, yet marginalized groups are often underrepresented (Fakkel et al., 2020). In this paper, when discussing marginalized groups, we refer to a heterogenous group which includes–but is not limited to–Black, Indigenous, and People of Color (BIPOC), individuals with bi-or multi-cultural origin, individuals from low socioeconomic status, individuals from the LHBTQIA + community, and individuals with disabilities or functional impairments. Although marginalized groups may differ per country and continent, and some of these groups may be considered marginalized predominantly in Europe and North America, the described groups still have historically been underrepresented in neuroscientific studies (Dotson and Duarte, 2020). Researchers generally attract and engage convenience samples, i.e., participants that have affinity with a specific research topic or are easy to contact and recruit. Convenience samples commonly do not reflect the heterogeneity of human populations. As a consequence, the underrepresentation of marginalized groups in neuroscientific studies is problematic for the generalizability of findings about (developmental) brain-behavior mechanisms, as well as for the validity, reliability, and reproducibility of results (Falk et al., 2013; Nielsen et al., 2017a; Dotson and Duarte, 2020). In turn, this may limit our general understanding of neurodevelopmental processes investigated in the population (Lewinn et al., 2017). Importantly, researchers should not solely focus on diversity; but also address inclusion and equity. Inclusion refers to the intentional process and effort to ensure that individuals with diverse identities can equally participate within an organization or group and that their contribution is equally valued by others (Tan, 2019). Equity refers to the leveling of the playing field for marginalized groups, the process of establishing access to the same opportunities and resources for all (Tan, 2019). Diverse research samples cannot be realized without committing to inclusive research and equity, since most issues regarding underrepresentation of marginalized groups are about unequal opportunities. In the present paper, we highlight three types of selection bias: (1) exclusion bias, (2) sampling bias and, (3) attrition bias in cohort studies. Second, we offer practical recommendations to minimize selection bias with a special focus on citizen science.

## Selection bias

The term selection bias encompasses the failure to select, attract or maintain a representative sample or study population (Hernán et al., 2004). Selection biases limit our possibilities to draw accurate conclusion from scientific findings, as there are systematic differences between the individual characteristics of the sample and the target population. Below, we discuss three forms of selection biases in developmental human neuroscience.

### Exclusion bias

Decades of studies in developmental cognitive neuroscience have greatly improved our understanding of a wide range of psychological processes and their neural underpinnings throughout development (Nelson and Bloom, 1997; Decety and Meyer, 2008). At the same time, these developmental processes were not always successfully measured among youth from marginalized groups, partly due to the selection of certain inclusion or exclusion criteria. Traditional rationales to exclude certain groups may be outdated and invalid, or systematically limit participation of marginalized individuals. For instance, specific neuroscientific tools and methodologies, including electroencephalography (EEG), functional near-infrared spectroscopy (fNIRS), skin conductance and eye tracking, often systematically exclude participants based on phenotypic differences, such as hair structure, skin pigmentation, pupil color (Webb et al., 2022). An example of this is how BIPOC youth with curly and tightly coiled hair have been excluded from EEG studies, due to the limited knowledge on ensuring good brain activity quality among diverse hairstyles or hair structures (Etienne et al., 2020; Choy et al., 2022). As a result, findings are often biased and difficult to generalize to BIPOC youth–limiting our understanding of their neurodevelopment–and how these individuals may be optimally supported during development (e.g., using prevention, intervention, and treatment).

These problems are often amplified by predominant reliance on group-based statistical comparisons in which neural measures are averaged across a group of homogenous participants to maximize statistical power (Willems et al., 2014)–despite evidence that grouping is often difficult and arbitrary as many population characteristics exist on a spectrum (i.e., show marked heterogeneity, or individual differences). In addition, how these characteristics are defined and operationalized in the first place widely differs between studies and countries (see Paus, 2010 for various examples on brain outcomes, and environmental factors like socioeconomic status). Hence, it is important to take steps to inclusively account for diversity and heterogeneity in our research samples, in operationalization, sampling strategies and data analysis.

### Sampling bias

Sampling bias may occur in studies when researcher do not properly select the study population (Nielsen et al., 2017a). Current recruitment strategies do not always allow us to successfully reach out to marginalized groups. Subsequently, systematic barriers prevent us from reaching diverse target groups from various marginalized groups (Habibi et al., 2015; Nielsen et al., 2017a). One of those barriers is the lack of accessibility to information and resources (Habibi et al., 2015). For example, individuals from lower socioeconomic backgrounds may have less access to financial and digital resources, which could prevent them from initial participation in neuroimaging studies (Jang and Vorderstrasse, 2019). Another example is how individuals with disabilities or functional impairment may face more difficulties when it comes to transportation to centers where neuroscientific studies are conducted (e.g., mobility issues, obstacles in public transport, or financial costs). A lack of diversity in research teams (Tzovara et al., 2021) may also limit recruitment amongst diverse groups of participants (Flores et al., 2017). Having representative scientists in research teams may result in feelings of familiarity and similarity among participants, which in turn may positively contribute to increased trust in science (Wallace and Bartlett, 2013; Flores et al., 2017). The importance of diversifying teams to promote equality was also evident in a study by Auelua-toomey and Roberts (2022). The authors showed that journals with diverse editorial boards were perceived more positively by both BIPOC and white graduate students than editorial boards without BIPOC members. Recently, the Organization for Human Brain Mapping (OHBM) has established a Diversity and Inclusivity Committee to promote the presence of underrepresented scientists and to create diverse role models in the field of neuroimaging [for a detailed overview of their code of conduct and their activities aimed at enhancing and fostering diversity, equity, and inclusion in academic teams see Tzovara et al. (2021)]. It is beyond the scope of this short paper to provide recommendations on how to diversify research teams in neuroscience, still it is important to acknowledge that having members of underrepresented groups is necessary for moving forward (Nielsen et al., 2017b; Harrington, 2021; Auelua-toomey and Roberts, 2022).

### Attrition bias

The third source causing potential selection bias is attrition. Longitudinal (neuroimaging) studies tend to end up with less representative research samples after each wave due to relatively high levels of attrition of participants from marginalized groups or low educated groups (Ewing et al., 2018). There is growing statistical literature on how to deal with missing data (e.g., multiple imputation methods) and attrition (e.g., inverse probability weighting) in longitudinal analyses. However, there is limited information on how to prevent systematic attrition marginalized groups in follow-up studies. To illustrate the loss of individuals from marginalized groups in cohort studies we highlight two neuroimaging studies, although attrition bias is common issue among most longitudinal studies. For example, in the Generation R Study, a population-based cohort study in Rotterdam, Netherlands the research sample became less diverse in terms of ethnicity and educational level with each wave, despite several efforts to keep youth form marginalized groups within the study. At the onset of the study, 48% of the participants were identified as Dutch, while this percentage increased to 55.8% 9 years later (Jaddoe et al., 2006; White et al., 2018). Researchers had invested in several efforts, including support for verbal translation of questionnaires in Turkish and Arabic by research assistants who even visited the participants at home (Jaddoe et al., 2008). Unfortunately, this was not enough to combat attrition among marginalized groups. Likewise, in the IMAGEN study, 17% of the parents had a low education level at baseline, while at the 5-year follow-up this was 13% (Modabbernia et al., 2021).

There may be multiple causes of this unfortunate loss of diversity, including logistical barriers (Nicholson et al., 2015; Flores et al., 2017; Raphael et al., 2017). Neuroimaging techniques, like Magnetic Resonance Imaging (MRI), can be time consuming. Some MRI sequences require laboratory visits of at least 3 h. Visit to research centers in general can be intensive, as behavioral and psychiatric assessments also tend to consume quite some time. Although this might not be a problem for some participants, it may prevent specific groups from participating multiple times. Additionally, studies have shown that adolescents from certain ethnically/culturally diverse groups and lower socioeconomic backgrounds tend to grow up in home environment with larger household, family or work responsibilities possibly making it more difficult for them to arrange free time for each assessment (Tseng, 2004; Sánchez et al., 2010).

## Connecting science to society

Bridging the gap between science and society allows for more representative, innovative and generalizable research, which may ultimately benefit healthcare practices, education and policy efforts (Ellis et al., 2021). Here, we provide some experience- and evidence-based recommendations to enhance diversity, equity, and inclusion in developmental human neuroscience through activities that are aimed at connecting science to society.

### Science communication and outreach

One way to connect science to society, specifically youth, is *via* science communication and outreach activities (Vollbrecht et al., 2019; Lichtenberg et al., 2020; a. Science communication can positively aid in building trust within communities, which in turn can have positive effects on dismantling selection biases (Saragosa-Harris et al., 2022). Prior research has shown that building trust within communities encourages participation among marginalized groups (Jang and Vorderstrasse, 2019; Nooner et al., 2021).

Science communication should be inclusive, and this requires expanding communication styles to other forms, such as such as writing blogs, making videos (with subtitles and preferable in multiple languages), and giving lectures and workshops at schools or community centers. For example, the authors of the present paper engage in science communication *via* social media platforms like Instagram, in which they share their scientific findings with youth and youth organizations. The variety of science communication methods is needed to enhance accessibility to information in marginalized groups and to let adolescents become familiar with neuroscience. Making neuroscientific findings accessible and understandable is not only essential in reaching out to marginalized groups, but also for society in general. Scientific discoveries belong to all of us and hence researchers should not only be open and transparent about their knowledge and findings toward each other, but also to the broader public (Vandenbroucke et al., 2021). Informing and educating young people about brain development and behavioral processes contributes to their understanding of developmental human neuroscience, and thus themselves. In addition, science communication and education can aid in making youth enthusiastic about science and academia (Tzovara et al., 2021). Enabling children from underrepresented groups to get familiar with (neuro) science is, one of the many actions in a chain of changes, needed to diversify academia.

In outreach programs scientists and/or students provide active learning experiences to adolescents and engage adolescents in science (Vollbrecht et al., 2019). Outreach programs are most common within the field of STEM (Science, Technology, Engineering and Mathematics), where the program aim is to attract a wider variety of students into STEM. However, some outreach programs still fail to reach adolescents from marginalized groups (Bultitude, 2014; Vollbrecht et al., 2019). The ABCD study has designed an outreach framework to raise awareness and promote sustainable support from different societal partners, including adolescents (Hoffman et al., 2018). Their framework follows four principles: (1) the identification and segmentation of target audience; (2) gaining support from community leaders and societal organizations; (3) the development and refinement from outreach materials disseminated *via* various platforms; and (4) feedback and evaluation of messaging and branding (Hoffman et al., 2018). Similar to the Generation R study (Jaddoe et al., 2008; Kooijman et al., 2016), the ABCD study maintains regular and dynamic contact with their participants *via* retention materials like newsletters, birthday cards, thank you presents, and reminders.

### Citizen science

A second method for connecting science to society is by taking the views and opinions of adolescents from diverse backgrounds actively into account through citizen science. Citizen science is a method in which adolescents are engaged as “citizens” in research projects rather than research participants (Te Brinke et al., 2022). By engaging in citizen science, researchers can learn from youth themselves. For example, what is the best way to contact them, what do they need to have access to longitudinal cohort studies, which barriers should be prioritized, and what do they think is needed for inclusive research? These are all questions for which adolescents from marginalized communities can give essential and valuable information, and thus aid researchers in tackling selection biases.

A crucial requirement for citizen science to work and to be beneficial for both society and science, is the establishment of equal researcher-community partnerships (Hoffman et al., 2018; Weng et al., 2020; Vandenbroucke et al., 2021). Engaging in researcher-community partnerships also entails giving youth partial responsibility and ownership. This will positively contribute to their sense of agency and will likely keep them involved and committed to project. In addition, citizen science should not only be beneficial for the researchers, but also for the adolescents within the community. Citizen science requires commitment from adolescents. Therefore, researchers should ask themselves: “what’s in it for adolescents? what can they get out of this commitment.” Although this may differ per individual or group, for most adolescents one of the requirements would be that that their ideas and perspectives will contribute to making impact. To ensure that adolescents feel heard and taken seriously when sharing their experiences and knowledge with researchers, it is crucial that researchers are transparent and open about how they incorporate the input from the community into their research. Additionally, involvement from communities, especially from marginalized groups, should also be reckoned and valued through financial compensation or by granting them a certificate.

For successful integration of citizen science initiatives, it is key that perspectives from adolescents, are included from the start of the research project (Vandenbroucke et al., 2021). Early inclusion of adolescents from marginalized groups in neuroscientific research (i.e., when writing the research proposal or setting up the research design) may aid in recognizing implicit biases that affect societal and scientific progress. Research projects should be more tailored to the needs, possibilities, and limitations of adolescents who participate in cohort studies (Jaddoe et al., 2006; Garavan et al., 2018; Hoffman et al., 2018; Nooner et al., 2021). We argue that having the right sampling and engagement strategies cannot be fulfilled without a sustainable researcher-community partnership, in which youth, youth workers, parents, teachers or communities are involved from the beginning of the research process (Weng et al., 2020; Saragosa-Harris et al., 2022). For instance, in the ABCD study, the researchers employed a probability sampling of schools as primary method for recruiting participants (Garavan et al., 2018). However, they also used additional strategies, such as outreach to summer activities and snowballing referral, whereby enrolled families would receive compensation for getting other families to participate in the study. This latter approach enables word-of mouth enrollment, where individuals can act as ambassadors of the study, and which is likely to have beneficial effects in enhancing trust among potential participants (Garavan et al., 2018).

All in all, recruitment of diverse and representative samples in neuroscientific studies requires a broad range of recruitment strategies, as the “one-size fit all” approach is vulnerable to selection biases. Recently, researchers recognize the scientific value of incorporating diverse perspectives from society into academia (Weng et al., 2020). As a result, research-community partnerships can lead to new innovative ideas, greater equity and societal impact and further scientific progress (Whitmore and Mills, 2021).

### Co-creation with societal partners

Co-creation is a specific form of citizen science were researchers and citizens collaborate in the development of a tool, measurement, or design. Within developmental psychology, researchers are gaining more experiencing in how to effectively work with youth at different stages of the empirical cycle, including developing measurement materials (Te Brinke et al., 2022). For instance, adolescents can actively engage in the creation of a new questionnaire or survey, by sharing ideas and providing feedback on the duration, types of questions or the language use. Although, co-creation may seem challenging within the field of developmental human neuroscience, it is still possible to collaboratively develop something with adolescents or other societal partners. Expertise may not always be fully present within the research teams, thus working together with societal partners with specific skills could be the solution. To highlight this, we use the example of how BIPOC individuals with curly and coiled hair structure, are systematically excluded from neuroscientific studies, such as EEG. The Biomechanics, Rehabilitation, and Interdisciplinary Neuroscience (BRaIN) lab at the University of Central Florida has developed an open-source guideline for including diverse hairstyles and hair structures in EEG research.^1^ Their guideline contains valuable information for both researchers and participants on hair preparation and hair care in EEG research. More importantly, they co-created this guideline in close collaboration with hair stylists from marginalized communities. This is an example of how societal partners can be of value in dismantling selection biases.

## Conclusion

In this short paper, we highlighted three selection biases in developmental human neuroscience studies that may influence the validity, reliability, and reproducibility of study results and thus limit our general understanding of neurodevelopmental processes. Further, we provided experience- and evidence-based recommendations to stimulate neuroscientists to enhance study population representativeness. For future research, we will need to get more insights in when its valuable to have diversity across homogeneous groups and when it is better to have diversity within one sample (i.e., heterogeneous sample). Representativeness and promoting participation of underrepresented groups can be achieved in both ways. Equal researcher-community partnerships and co-creation of research projects with youth from marginalized groups are of great added value to tackle systemic barriers. By connecting science to society, we have the opportunity to both transfer scientific findings to youth, as well as to bring new perspectives and knowledge from society back to the lab, especially from individuals from marginalized groups who have historically been left out.

## Data availability statement

The original contributions presented in this study are included in the article/supplementary material, further inquiries can be directed to the corresponding author.

## Author contributions

KG: conceptualization, investigation, and writing – original draft and review and editing. IV: investigation and writing – original draft and review and editing. LT and FR: investigation and writing – original draft. RC: conceptualization. HE conceptualization, review and editing, and supervision. All authors contributed to the article and approved the submitted version.

## Footnotes

1
https://hellobrainlab.com/research/eeg-hair-project/
